# Effect of immersive virtual mirror visual feedback on Mu suppression and coherence in motor and parietal cortex in stroke

**DOI:** 10.1038/s41598-023-38749-8

**Published:** 2023-08-02

**Authors:** Won Kee Chang, Hyunmi Lim, Seo Hyun Park, Chaiyoung Lim, Nam-Jong Paik, Won-Seok Kim, Jeonghun Ku

**Affiliations:** 1 Department of Rehabilitation Medicine, Seoul National University College of Medicine, Seoul National University Bundang Hospital, Seongnam, Republic of Korea; 2 Department of Biomedical Engineering, College of Medicine, Keimyung University, 1095, Dalgubeol-daero, Dalseo-gu, Daegu, Republic of Korea; 3 Bundang Rusk Rehabilitation Speciality Hospital, Seongnam, Republic of Korea; 4 Department of Biomedical Engineering, School of Medicine, Keimyung University, Daegu, Republic of Korea

**Keywords:** Neurological disorders, Neuroscience

## Abstract

We investigated the activation pattern of the motor cortex (M1) and parietal cortex during immersive virtual reality (VR)-based mirror visual feedback (MVF) of the upper limb in 14 patients with chronic stroke and severe upper limb hemiparesis and in 21 healthy controls. Participants performed wrist extension with unaffected wrists (dominant side in controls). In the MVF condition, movement of the affected hand was synchronized with that of the unaffected hand. In the no-MVF condition, only the movement of unaffected hand was shown. Mu suppression in bilateral M1 and parietal cortex and mu coherence were analyzed. In patients with stroke, MVF induced significant mu suppression in both the ipsilesional M1 and parietal lobes (p = 0.006 and p = 0.009, respectively), while mu suppression was observed in the bilateral M1 (p = 0.003 for ipsilesional and p = 0.041 for contralesional M1, respectively) and contralesional parietal lobes in the controls (p = 0.036). The ipsilesional mu coherence between the M1 and parietal cortex in patients with stroke was stronger than controls, regardless of MVF condition (p < 0.001), while mu coherence between interhemispheric M1 cortices was significantly weaker in patients with stroke (p = 0.032). Our findings provide evidence of the neural mechanism of MVF using immersive VR in patients with stroke.

## Introduction

Upper limb hemiparesis is one of the most common post-stroke impairments associated with activity limitation and quality of life^1,2^. Recovery of upper limb function is expected after stroke because of the spontaneous recovery and the effect of task-based conventional rehabilitation^3,4^. Still, recovery in patients with severe stroke with extensive corticospinal tract involvements is usually limited^5^. Therefore, effective restorative therapeutic approaches combined with conventional occupational therapy are required for patients with stroke with severe upper limb hemiparesis^6^.

Mirror therapy provides mirror visual feedback (MVF) of the unimpaired limb, making the patients feel the illusion of both limbs moving synchronously without impairment^7^. Mirror therapy has demonstrated moderate-quality evidence for its moderate-size beneficial effects on motor recovery in the upper limb after stroke when combined with conventional rehabilitation in a recent meta-analysis^8^. In patients with severe hemiparesis, conventional task-oriented occupational therapy is difficult to provide, and mirror therapy is a useful rehabilitation approach^9^.

Despite the clinical use and evidence of mirror therapy in upper limb rehabilitation after stroke, the underlying mechanism of MVF is inconclusive. Popular theories include the enhancement of motor imagery by MVF, which leads to the activation of a mirror neuron system composed of the premotor cortex, supplementary motor area, inferior frontal gyrus, and inferior parietal lobule^10–12^. However, in the first study of MVF in patients with stroke using functional magnetic resonance imaging (fMRI), the MVF did not induce mirror neuron system activation but activated the precuneus and posterior cingulate cortex, which are associated with awareness of the self and spatial attention^13^. In a recent systematic review of the effect of MVF on the brain, little evidence was found that MVF activates the mirror neuron system. Despite this, MVF activated the right dorsolateral prefrontal, contralateral sensory, and ipsilateral superior posterior parietal cortices, which are associated with attention and cognitive control^14^. Therefore, in patients with stroke, the traditional hypothesis regarding the role of the mirror neuron system in MVF is conflicting.

In previous studies using fMRI, immersive virtual reality (VR) has been used to determine the mechanism of MVF^15–18^. Immersive VR is useful for providing the illusion that only the paretic upper limb is moving in an enriched environment. Weber et al. recently demonstrated the feasibility of immersive VR mirror therapy in patients with chronic stroke^19^. Understanding the cortical mechanism of VR-based mirror therapy is vital for designing an effective treatment protocol and combining effective adjunctive modalities such as neuromodulation. Interestingly, a series of previous studies on immersive VR in stroke demonstrated ipsi- or contralesional parietal activation owing to the increase in self-awareness of the paretic hand, which leads to an increase in ipsilesional primary motor cortex (M1) activation^16,17^. However, these results may not be generalized to patients with severe stroke since Tai et al.^20^ reported a different neurophysiological response to conventional mirror therapy in patients with severe paresis. The patients included in previous studies had mild to moderate hand weakness and stages 3 to 7 according to the Chedoke-McMaster Rating Scale for the hand^16,17^. Mirror therapy may be more useful in patients who cannot participate in conventional task-based occupational therapy with severe hand weakness^21^. In addition, the role of neuroplasticity differs according to the degree of cortical spinal tract involvement in stroke. For instance, the contralesional hemisphere may play a more important role, while interhemispheric inhibition is less important in patients with severe corticospinal tract damage^22^.

Mu wave, an 8–13 Hz frequency rhythm measured with electroencephalography (EEG) which arise from the sensorimotor area, has been extensively studied to investigate the activities of the human mirror neuron system^23^. Mu suppression, an index for measuring the degree of activation of the sensorimotor cortices^24^, and mu coherence, an index to assess the strength of connectivity between the sensorimotor cortices^25^, are widely used indices to measure brain activities during sensorimotor tasks.

We designed this study to investigate the cortical mechanism of immersive virtual MVF in patients with chronic stroke with severe hand hemiparesis. We hypothesized that immersive virtual MVF would induce cortical activation in the ipsilesional M1 and parietal cortex. EEG was used to investigate the changes in mu suppression in the M1 and parietal cortex by virtual MVF in healthy controls and patients with stroke. As a marker for connectivity, mu coherence between the ipsilesional M1 and parietal cortex was also analyzed using EEG.

## Methods

### Participants

Fourteen patients with severe unilateral hand paresis (1 to 3 in Brunnstrom stage of hand)^26^ after their first-ever ischemic or chronic hemorrhagic stroke (> 3 months after onset) were recruited. Patients with a previous injury in the central nervous system, who had cognitive deficit (defined as a Mini Mental Status Examination score < 16), or who could not understand and follow experiment instructions were excluded. Twenty healthy right-handed adults with no previous history of diseases involving the central nervous system or any upper limb weakness or injuries were included.

### Experiment design and task description

**Figure 1:**
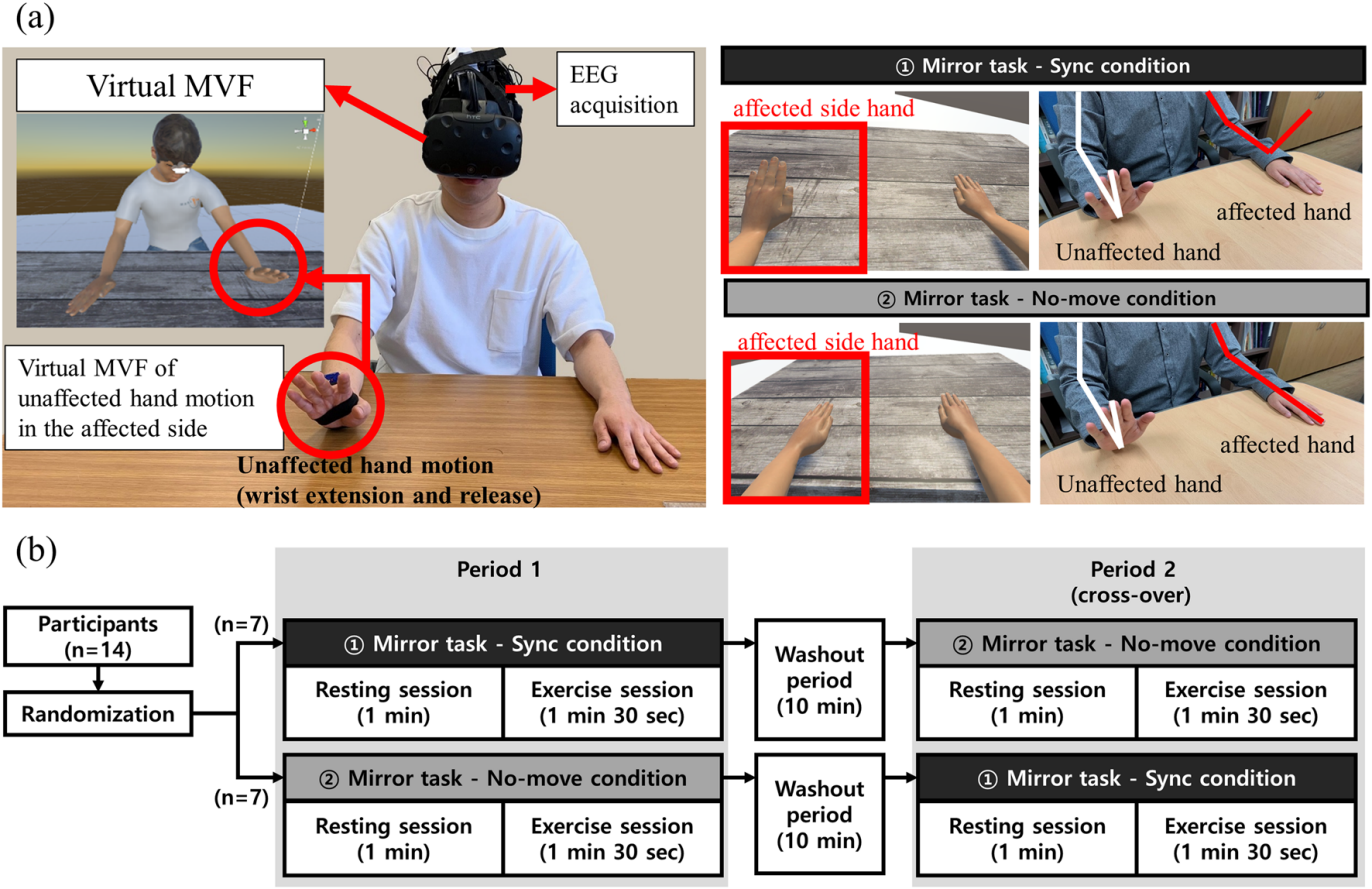
Schematic drawing of the experiment. (**a**) Experimental configuration and condition description (**b**) Experimental design.

The experiment consisted of two sessions of resting for 60 s and exercising for 90 s (Fig. 1b). In the resting session, a white fixation cross with a black background appeared and was fixated for 60 s without upper limb movement, followed by an exercise session. In the exercise session, participants were instructed to extend and release their unaffected wrist (dominant in controls) at a frequency of 0.25 ~ 0.3 Hz and not to move their affected upper limbs (non-dominant in controls) while staring at the virtual hand on the opposite side for 90 s. There were two conditions in the exercise session: (1) MVF condition, in which the opposite virtual hand was synchronously moved with the real unaffected hand movements so that one could feel as if their real affected hands were moved, even when the affected hand was not moved at all, and (2) No-MVF condition, in which the virtual hand was not moved regardless of the movement of the unaffected hand. Participants performed two independent experiments in a day to experience both conditions; however, the order of the two conditions was randomly allocated for each participant. The two experiments were performed with a wash-out period of 10 min.

### Experimental procedure

The participants were asked to sit in front of the experimental table by placing their upper limbs on the table comfortably by placing the left and right hands at a distance of 25 cm from the midline and 40 cm forward from the body. The motion sensor was attached to the back of the hand to be moved in this experiment (unaffected hand for patients and dominant hand (right) for the healthy control). They wore an EEG cap, and calibration steps were conducted for EEG data acquisition. Subsequently, a head-mounted display (HTC VIVE, HTC corporation, Taoyuan City, Taiwan) was used to enable them to experience an immersive virtual environment. In the virtual environment, there was a table and an avatar with a posture similar to that of the participants by placing their upper limbs on the table. We tried to fit the avatar's posture to that of the participant’s as close as possible in size and position so that they could see the virtual hands aligned with their own. After completing the preparation, they were instructed to follow the instructions on the head-mounted display and participate in the experiment (Fig. 1a).

### EEG acquisition and parameters extraction

EEG data were collected using different devices in patients and healthy controls. A thirty-two channel EEG system (Brain Products, GmbH, Germany) was used for patients, and a DSI-24 (Wearable Sensing) EEG device with 19 dry electrodes and a 128 Hz sampling rate was used for healthy controls. The electrodes were arranged according to the International 10–20 system. The quality of the EEG signal was verified before the experiment.

The recorded EEG was analyzed to examine the brain activity during the task, with the experience of the virtual mirror visual feedback of the upper limb being related to the desynchronization and coherence of the mu band (8–13 Hz). In this study, we analyzed EEG data from C3, C4, P3, and P4 because we were only interested in the involvement of the motor and parietal cortices. We examined the mu suppression in these channels. The coherence of C3-P3 and C4-P4 for the interaction between the motor and parietal cortex in each hemisphere was analyzed.

For preprocessing, band-pass filtering with a 4–30 Hz filter, artifact subspace reconstruction (ASR)^27^, and independent component analysis methods^28^ were applied to the signal to eliminate unwanted noise signals, such as physiological noise components, and then the filtered signal was segmented with a 1 s epoch using a Hamming window function. Epochs with an average amplitude exceeding a threshold (70 µV) were excluded. With each epoch, the mu-band power and coherence strength were calculated, revealing a series of values that would then be averaged.

The Fast Fourier transformation of each surviving epoch was conducted for mu-band power extraction. The values of the mu-band power from each epoch were averaged, and the average value was used to represent the mu-band power during the task. The mu-band power during the task was compared with the mu-band power during rest, which was obtained before the task, using a log ratio. This means that a positive or negative ratio would represent an increase or decrease, respectively, of the mu-band power compared to that of the resting state, while the log ratio value would be zero if there were no changes in power compared to that of the resting state.

For extracting the strength of mu coherence between channels, the magnitude squared coherence (MSC) was computed with the 'mscohere' function in the Matlab version2014a (Mathworks, Natick, MA, USA). The MSC was estimated according to the formula $\mathrm{Coh }= {\mathrm{S}}_{\mathrm{xy}}^{2} / ({\mathrm{S}}_{\mathrm{xx}} \times {\mathrm{S}}_{\mathrm{yy}})$, where S_xy_, S_xx_, and S_yy_ are the averaged cross-spectrum and auto-spectrum estimates for the signals from channels x and y, respectively. This formula calculates Coh as a function of frequency and takes values between 0 and 1, which indicates how well a signal x(t) corresponds to a signal y(t) at each frequency. The value of 0 at a given frequency indicates that the two signals are completely independent at this frequency, whereas the value of 1 corresponds to two signals with identical frequency components. Values ranging between 0 and 1 indicate the number of common parts of the signals at this frequency. We computed the mean Coh values within the mu-band frequencies in this study.

### Statistical analysis

Mu suppression in the M1 and parietal cortex in each hemisphere and mu coherence between two ipsilateral cortical areas (M1-Parietal cortex) were used for the analysis. Two-way repeated Measures ANOVA, MVF condition (MVF vs no-MVF) × Group (stroke vs control) × Hemisphere (ipsilesional (ipsilateral to the moving dominant hand in controls) vs contralesional (contralateral to the moving dominant hand in controls)) was used. For the posthoc test to compare the effect between MVF and no MVF on mu suppression and mu coherence in four conditions by group and hemisphere, we generated contrasts to compare the marginal means between the four conditions, and the Bonferroni corrected *P*-values were calculated. In addition, we performed the same posthoc tests to compare the mu coherence according to group and hemisphere in the following two situations with four conditions in each situation: (1) stroke vs control in four conditions by MVF and hemisphere; (2) ipsilesional vs contralesional in four conditions by MVF and group. Partial eta-squared (η^2^) value was calculated in the variables with significant effect on EEG indices. Statistical significance was set at P < 0.05. All statistical analyses were conducted using R 4.1.0 (R Foundation for Statistical Computing, Vienna, Austria).

### Ethics approval and consent to participate

All participants provided written informed consent after receiving detailed information about the study. The research protocol was conducted in accordance with the regulatory standards of Good Clinical Practice and the Declaration of Helsinki^29^. The Institutional Review Board approved the study protocol of the Seoul National University Bundang Hospital (B-1902/523-301) and the Institutional Review Board of the Keimyung University Dongsan Hospital (IRB number:2019-03-017-001).

## Results

Fourteen patients with stroke (age: 56.0 ± 13.7, M: F = 9:5) were recruited. Table 1 shows the demographic information of patients with stroke. Twenty volunteers (6 males, 14 females) aged 20–28 years (mean age: 22.4 ± 2.41 years) participated in the experiment.

**Table 1:** Demographic information of patients with stroke.

ID	Age	Sex	Stroke type	Stroke location	Hemiparetic side	Time since stroke onset (months)	Brunnstrom-stage (arm)	Brunnstrom-stage (hand)
1	25	M	Hemorrhagic	Basal ganglia	Left	12	3	3
2	71	M	Ischemic	Corona radiata	Right	4	3	3
3	49	M	Hemorrhagic	Basal ganglia, corona radiata	Right	12	3	3
4	46	M	Hemorrhagic	Basal ganglia	Right	4	2	2
5	52	F	Hemorrhagic	Fronto-temporal	Left	13	4	3
6	75	F	Ischemic	Basal ganglia, corona radiata	Left	14	2	2
7	70	F	Ischemic	Middle cerebral artery territory	Left	9	2	2
8	69	M	Ischemic	Basal ganglia, corona radiata	Left	9	3	3
9	42	M	Hemorrhagic	Basal ganglia	Left	8	3	3
10	49	M	Hemorrhagic	Fronto-parietal	Right	7	3	3
11	55	F	Hemorrhagic	Basal ganglia	Left	7	3	3
12	54	M	Hemorrhagic	Basal ganglia	Left	16	3	3
13	61	F	Hemorrhagic	Corona radiata	Left	5	4	3
14	66	M	Hemorrhagic	Basal ganglia, thalamus	Right	13	4	3

### Mu suppression in the M1 and parietal cortex

The mixed-effects ANOVA on mu suppression (Table 2) showed a significant main effect between the two conditions (MVF vs no-MVF) on M1 (p < 0.001, η^2^ = 0.093) and the parietal cortex (p = 0.003, η^2^ = 0.062), and there was a significant interaction effect among the three factors (MVF condition * Group * Hemisphere) on the parietal cortex (p = 0.006, η^2^ = 0.014). Other comparisons did not reveal any significant effects.

**Table 2:** Results of mixed effects ANOVA on Mu suppression.

Factors	M1		Parietal cortex	
	F	*P*	F	*P*
MVF condition (MVF vs no-MVF)	25.489	< 0.001	10.522	0.003
Group (stroke vs healthy controls)	1.205	0.281	3.742	0.062
Hemisphere^a^	0.078	0.782	0.350	0.558
MVF condition × group	0.025	0.875	0.609	0.441
MVF condition × hemisphere	1.701	0.201	0.006	0.939
Hemisphere × group	0.056	0.815	0.013	0.910
MVF condition × group × hemisphere	0.042	0.839	8.513	0.006

*MVF* mirror visual feedback.

^a^Ipsilesional (ipsilateral to the moving hand in controls) vs contralesional (contralateral to the moving hand in controls).

**Figure 2:**
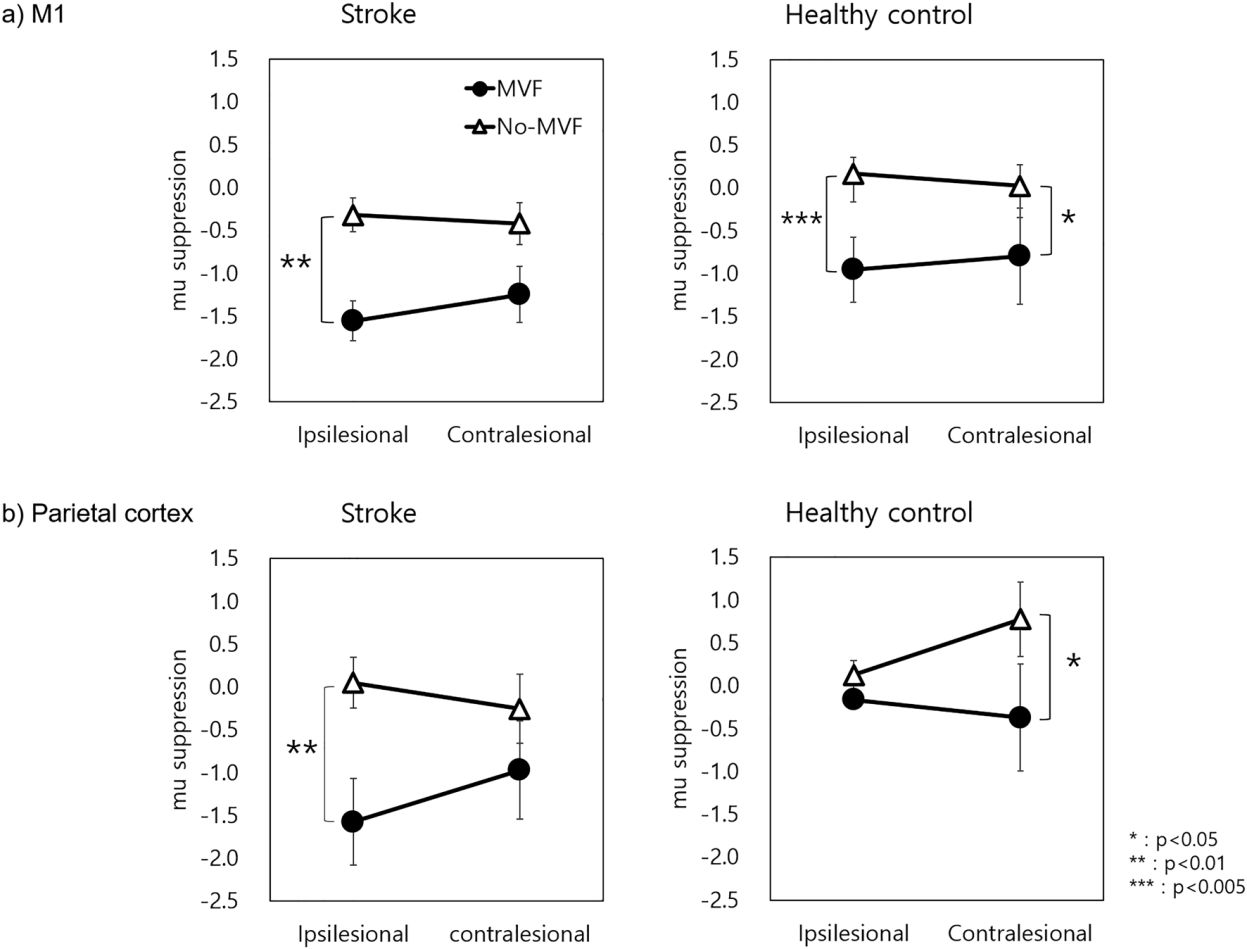
Mu suppression in M1 and parietal cortex. The amount of mu suppression of the ipsilesional and contralesional side in (**a**) the M1 and (**b**) the parietal cortex for patients with stroke and for healthy controls. In the healthy controls, the ipsilesional side indicates that it is ipsilateral to the moving hand and the inverse is true for the contralesional side. *p < 0.05, **p < 0.01 and ***p < 0.005.

The mu suppression values for MVF and no-MVF in the ipsilesional M1 were − 0.953 ± 1.699 and 0.166 ± 1.470 for healthy controls and − 1.555 ± 0.870 and − 0.317 ± 0.730 for patients with stroke, respectively (Fig. 2a). Both groups showed stronger mu suppression in the MVF than in the no-MVF in the ipsilesional M1 (p = 0.003 and p = 0.006 for healthy controls and patients with stroke, respectively). Meanwhile, those for the contralesional M1 region were − 0.795 ± 2.527 and 0.025 ± 1.671 for healthy controls, and − 1.246 ± 1.230 and − 0.419 ± 0.924 for patients with stroke in the MVF and no-MVF groups, respectively, in which only healthy controls showed significantly stronger suppression in the MVF group than in the no-MVF group (p = 0.041).

The mu suppression values in the ipsilesional parietal cortices were − 0.162 ± 0.428 and − 1.574 ± 1.902 for healthy controls and patients with stroke, respectively, in the MVF group, and 0.125 ± 0.773 and 0.050 ± 1.113 for healthy controls and patients with stroke, respectively, in the no-MVF group (Fig. 2b). In addition, the mu suppression values in the contralesional parietal cortex were − 0.370 ± 2.809 for healthy controls and − 0.971 ± 2.140 for patients with stroke in the MVF condition, and 0.776 ± 1.944 for the healthy control and − 0.254 ± 1.496 for patients with stroke in the no-MVF condition. In patients with stroke, mu suppression in the ipsilesional parietal cortex showed stronger suppression in the MVF (p = 0.009). In contrast, in healthy controls, mu suppression in the contralesional parietal cortex was more suppressed in the MVF (p = 0.036). There was no significant effect of stroke type (infarction vs hemorrhage) or time since onset on mu suppression in both the M1 and parietal cortex and mu coherence.

For mu coherence between the ipsilateral M1 and parietal cortex (IntraMPC), the mixed effect ANOVA (Table 3) showed significant main effects in the MVF condition (p = 0.001, η^2^ = 0.002), group (p < 0.001, η^2^ = 0.338), and hemisphere (p < 0.001, η^2^ = 0.214), and there were significant interaction effects in the MVF condition * group (p = 0.016, η^2^ = 0.001) and Hemisphere * Group (p < 0.001, η^2^ = 0.068).

**Table 3:** Results of mixed effects ANOVA on Mu coherence.

Factors	Mu coherence between ipsilateral M1-parietal cortex	
	F	*P*
MVF condition (MVF vs no-MVF)	12.171	0.001
Group (stroke vs healthy controls)	42.996	< 0.001
Hemisphere^a^	47.487	< 0.001
MVF condition × group	6.501	0.016
MVF condition × hemisphere	0.444	0.510
Hemisphere × group	19.196	< 0.001
MVF condition × group × hemisphere	1.281	0.266

*MVF* mirror visual feedback.

^a^Ipsilesional (ipsilateral to the moving hand in controls) vs contralesional (contralateral to the moving hand in controls).

**Figure 3:**
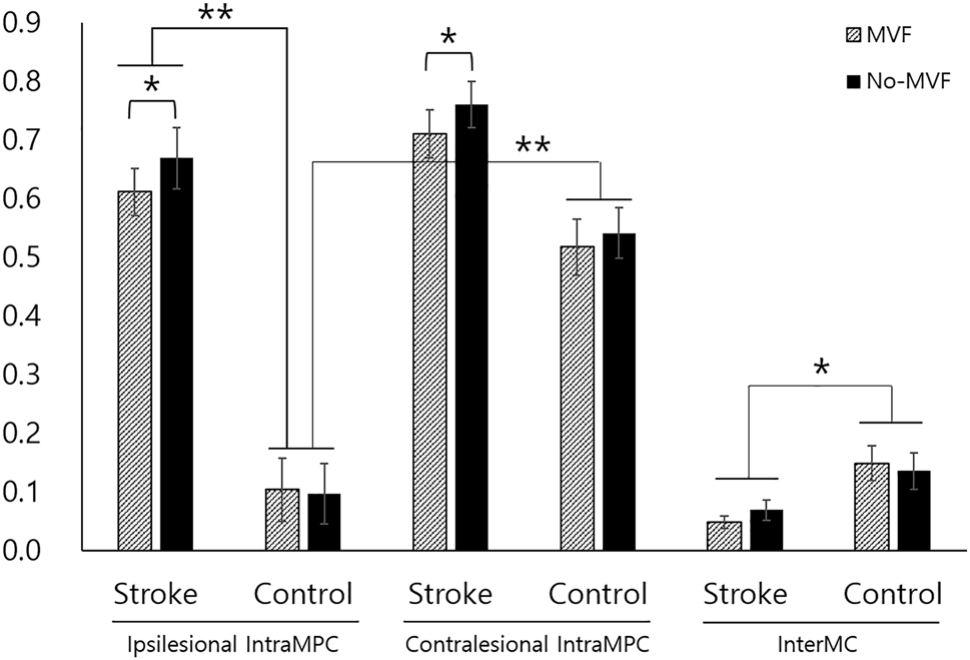
Mu coherence between ipsilateral M1 and parietal cortex (IntraMPC). Mu coherence between ipsilateral M1 and parietal cortex (IntraMPC) in the ipsi-, contralesional hemisphere and interhemispheric M1 (InterMC). *p < 0.05 and **p < 0.001.

Intra-MPC in patients with stroke was stronger in no-MVF than in MVF in both ipsilesional (p = 0.013) and contralesional hemispheres (p = 0.041), while no difference was observed in healthy controls (Fig. 3).

IntraMPC in the ipsilesional hemisphere was stronger in patients with stroke than in healthy controls, regardless of the MVF condition (p < 0.001). At the same time, there was no significant difference in the contralesional hemisphere between the stroke and control groups. In addition, IntraMPC was stronger in the contralesional hemisphere (contralateral to the moving hand) than in the ipsilesional hemisphere, regardless of the MVF condition. At the same time, patients with stroke did not show any differences according to the hemisphere side.

Interhemispheric mu coherence between the M1 cortices (InterMC) was significantly stronger in healthy controls than in patients with stroke (p = 0.032). At the same time, the mixed effect ANOVA model revealed no significant effect of MVF on IntraMC and IntraPC in patients with stroke and healthy controls. The individual subjects' data can be found in the Supplementary Information.

## Discussion

In this study, we examined the differences in M1 and parietal lobe neural activity during virtual MVF and no-MVF conditions by measuring the degree of mu suppression and coherence between them. Our results show that virtual MVF elicits significant mu suppression in the ipsilesional M1 and parietal lobe in patients with chronic stroke. In contrast, significant mu suppression was observed in the bilateral M1 and contralesional parietal lobes in healthy participants. IntraMPC in the ipsilesional hemisphere was significantly higher in patients with stroke than in healthy controls, whereas InterMC was stronger in healthy controls. The MVF setting showed a significant decrease in mu coherence in the ipsilesional and contralesional hemispheres in patients with stroke, while no significant effect was observed in healthy controls.

Activation of the ipsilesional M1 and parietal lobe by MVF has been previously reported in patients with stroke^13,15,16,30^. Our study results also demonstrate significant mu suppression in the ipsilesional M1 and parietal lobe in patients with stroke, inferring that MVF using immersive VR elicits modulation of the ipsilesional sensorimotor network. The discrepancy between visual and proprioception is considered a vital point of the underlying neural mechanism of MVF^31^; Fritsch et al. reported that the parietal lobe acts as a critical region of visuomotor transformation by relaying visuospatial information to the primary motor cortex^32^. Thus, activation of the ipsilesional sensorimotor network supports the underlying neuro mechanism of the therapeutic effect of MVF in restoring motor deficits in patients with stroke^14^, which normalizes the altered interhemispheric balance between sensorimotor cortices, a pivotal finding in the recovery of motor function in patients with stroke^33,34^.

Mu suppression in the bilateral M1 and contralesional parietal lobes was observed in the healthy controls in our study. Zhang et al. reported bilateral activation of the bilateral motor cortex during MVF in healthy controls using EEG^35^, and Saleh et al. reported connectivity between the contralesional parietal lobe and ipsilesional M1^17^. Our findings align with those reports that support the hypothesis of bilateral sensorimotor network activation by MVF. However, other reports have reported that MVF elicits ipsilesional activation of M1^36–38^ and ipsilesional or bilateral parietal lobes in healthy controls^18,39^, similar to that in patients with stroke. The discrepancy may lie in the type of MVF (VR mirror vs real mirror) and neuroimaging tool (fMRI vs EEG); however, this remains unclear.

In our study, ipsilesional IntraMPC was significantly stronger, whereas InterMC was significantly weaker in the patient group. Wang et al.^40^ demonstrated increased resting intrahemispheric connectivity in the sensorimotor network in patients with chronic subcortical stroke. Zhang et al.^41^ demonstrated increased functional connectivity between the ipsilesional M1 and parietal lobe with decreased interhemispheric M1 connectivity. Inferring from these studies, the increased IntraMPC in the patients with stroke in our study may be a result of compensatory remodeling of functional connectivity in the sensorimotor network after stroke. However, the relationship between intrahemispheric sensorimotor connectivity and motor recovery after a stroke remains unclear. Wu et al.^42^ reported that motor gain in patients with stroke is negatively related to ipsilesional M1-parietal connectivity, while Tsuchimoto et al.^43^ reported increased ipsilesional sensorimotor network connectivity is associated with motor exercise using neurofeedback after stroke. Since our study did not include longitudinal changes in connectivity in response to MVF, the relationship between intraMPC, interMC, and the degree of motor recovery elicited by MVF still needs to be examined.

The effect of time since onset on mu coherence in patients with stroke needs further exploration since remodeling of functional connectivity can affect mu coherence. In our study, there was no significant effect of time since onset and this may be attributed to the characteristics of the participants of our study. The participants with stroke in this study were chronic patients with stroke onset of 4 to 16 months. Tombari et al.^44^ have reported that changes in neural activation are stabilized between 4 and 12 months after stroke. Giaquinto et al.^45^ have also reported that changes in EEG indices occur in the first 3 months. Moreover, Ward et al.^33^ and Guggisber et al.^46^ have reported that changes in functional connectivity are related to the degree of motor recovery in the chronic stroke phase and the patients in our study had homogenous characteristics regarding severe motor impairment.

Both the intraMPC and interMC decreased during MVF in the patients with stroke in this study, although the effect size was small (η^2^ = 0.002)^47^. Although there is no report on measured mu coherence during MVF in patients with stroke, the relationship between the mu coherence and the activation of sensorimotor network have been reported incongruently. Kim and Cho have reported that action observation, a task that activates the mirror neuron system, decreases both intrahemispheric and interhemispheric coherence of high alpha wave^48^, while van der Helden et al. have reported increased mu coherence during action observation^49^. Further research on this issue is required to conclude the relationship between mu coherence and activation of sensorimotor cortex.

In this study, we reported mu-wave suppression in the ipsilesional M1 and parietal lobe elicited by MVF in patients with stroke with severe upper-limb hemiparesis (hand Brunnstrom stage 1–3). This is notable since most previous studies on MVF included patients with mild to moderate-severity stroke^13,16,20,34^. Our data showed that MVF activated the ipsilesional sensorimotor network in patients with stroke with severe paresis, in congruence with Wang et al.^15^ They reported ipsilesional activation of the precuneus with fMRI in patients with stroke with severe hemiparesis. Considering that MVF is regarded as a therapeutic approach that can be used in patients with stroke with severe hemiparesis^50^, our findings will add additional neurophysiological grounds for using MVF in the severely paralyzed patient population with stroke^9^.

The use of immersive VR was another notable feature of our study. Mirror therapy using immersive VR has several advantages over conventional mirror therapy. Conventional mirror therapy is usually performed with simple tasks such as flexion/extension of the wrist; however, the use of immersive VR can enable the participants to execute more complicated tasks, such as activities of life, in an enriched VR environment^19^ and also bear elements of gamification^51^, which could provide beneficial effects on functional improvement in patients with stroke. Furthermore, mirror conditions using immersive VR are reported to activate the ipsilesional corticospinal tract more strongly than conventional mirroring^52^, and immersive VR has the chance to elicit a stronger therapeutic effect. However, further investigations are warranted to confirm this hypothesis.

The study has some potential limitations that need to be mentioned. First, the age of the healthy participants was significantly lower than that of patients with stroke. The relationship between age and the degree of cortical activation varies among reports. Wang et al.^15^ reported a negative correlation between age and cortical activation in healthy participants, while Rossiter et al.^34^ found no significant relationship between age and degree of beta desynchronization. Unfortunately, our results cannot provide information on the relationship between age and degree of cortical activation due to the different ages of the two groups, nor can we conclude whether the age difference between the two groups has affected the degree of sensorimotor network activation. However, even if a negative correlation between age and degree of cortical activation exists, MVF using immersive VR still induces sensorimotor network activation in older stroke participants. Second, the EEG acquisition systems used in the patients with stroke and the healthy participants were different, which is a limitation of this study. However, the same data processing technique was applied to the two EEG systems and the EEG indices we used in this study (mu suppression and mu coherence) are relative values compared to resting status^30^. Moreover, it has been reported that these EEG indices are valid across different EEG systems^53,54^. Third, the dosage of MVF was brief and insufficient to elicit clinical effect. The experiment applied 90 s of MVF, which is a shorter time compared to the clinical practice dosage of MVF^8^. However, the dosage of MVF in this experiment was sufficient to measure the neurophysiological effect of MVF, according to the previous studies^16,18,55^, but was too short to elicit clinical changes in the patients. Further study designs combining both shorter dosages of MVF to measure neurophysiological effects and longer dosages of MVF to measure clinical changes will provide a comprehensive understanding on the effect of MVF. Additionally, the sample size was relatively small, and a larger number of participants would lead to more robust conclusions. Lastly, a comparison of MVF using a real mirror, as well as neurophysiological parameters such as measurement of corticospinal excitability, hinders the conclusion of the effect of immersive VR on the neural mechanism of MVF. Further studies, including measuring various neurophysiological parameters compared to real mirrors, are required to address this issue.

## Conclusions

MVF using immersive VR elicits mu suppression in the ipsilesional M1 and parietal lobe in patients with stroke with severe hemiparesis, implying the activation of the ipsilesional sensorimotor network. Our findings provide evidence of the neural mechanism of MVF using immersive VR in patients with stroke.

## Supplementary Information

Supplementary Information.

## Data Availability

Pseudonymized data supporting this study’s findings are available from the corresponding author, Pf. Won-Seok Kim, or the IRB of Seoul National University Bundang Hospital (snubhirb@gmail.com), upon reasonable request, subsequent approval from the local IRB, and completion of a legal data-sharing agreement.

## Abbreviations

MVF: Mirror visual feedback
fMRI: Functional magnetic resonance imaging
VR: Virtual reality
M1: Primary motor cortex
EEG: Electroencephalography
MSC: Magnitude squared coherence
IntraMPC: Intrahemispheric M1-Parietal cortex mu coherence
InterMC: Interhemispheric M1 mu coherence
